# The association between myopia and mental health among Chinese children in primary and secondary school: a cross-sectional study

**DOI:** 10.3389/fpubh.2025.1598790

**Published:** 2025-06-03

**Authors:** Shijie Yu, Hongpo Yin, Wei Sun, Ruosong Yang, Ronghua Lai, Yushan Yu, Hongsheng Bi, Jianfeng Wu

**Affiliations:** ^1^Medical College of Optometry and Ophthalmology, Shandong University of Traditional Chinese Medicine, Jinan, Shandong, China; ^2^Affiliated Eye Hospital of Shandong University of Traditional Chinese Medicine, Jinan, Shandong, China; ^3^Department of Public Health and Primary Care, Faculty of Medicine and Health Sciences, International Centre for Reproductive Health, Ghent University, Ghent, Belgium; ^4^Shandong Provincial Key Laboratory of Integrated Traditional Chinese and Western Medicine for Prevention and Therapy of Ocular Diseases, Shandong Provincial Clinical Medical Research Center of Optometry and Adolescent Low Vision Prevention and Control, Shandong Engineering Technology Research Center of Visual Intelligence, Jinan, Shandong, China

**Keywords:** myopia, mental health, China, children, strengths and difficulties questionnaire

## Abstract

**Objective:**

This study aimed to investigate the association between myopia and mental health among Chinese children in primary and secondary school.

**Methods:**

This study employed a cross-sectional study design, including 1,640 children and their parents, via an online survey. The children underwent routine eye examinations including cycloplegic autorefraction. Multivariable linear and logistic regression models were used to examine the association between myopia and mental health. Multivariable linear regression analyses were performed in distinct subgroups.

**Results:**

Among all children, 836 children (50.98%) were boys; the mean age was 9.39 ± 2.22 years old. A decline in visual acuity last year was associated with a higher strengths and difficulties questionnaire (SDQ) total difficulties score (β = 0.564, 95% CI: 0.064, 1.064) and an increased risk of borderline mental health problems (OR = 1.863, 95% CI: 1.135, 3.057). Abnormal mental health problems were more likely to occur in myopic children than emmetropic children (OR = 3.601, 95% CI: 1.070, 12.456). The risk of abnormal mental health problems decreased with an increase in number of correctly identified the early treatment diabetic retinopathy study (ETDRS) markers (OR = 0.964, 95% CI: 0.933, 0.997). In grades 3-4, myopic children had a higher SDQ total difficulties score compared with emmetropic children (β = 1.065, 95% CI: 0.200, 1.929), and children with a decline in visual acuity last year had a higher SDQ total difficulties score than those without decline (β = 0.981, 95% CI: 0.011, 1.951).

**Conclusion:**

Myopic children, especially in grades 3-4, were more susceptible to mental health problems than emmetropic peers. This shows the importance of early targeted counseling for mental health in myopic children.

## 1 Introduction

With the surge in the global prevalence of myopia, the mental health of myopic population becomes an area of critical concern. People with visual impairment can experience mental problems (1). Myopia is a type of refractive error in which light entering the eye focuses in front of the retina parallel to the visual axis when eye accommodation is relaxed (2). In China, the overall prevalence of myopia among children and adolescents has reached nearly 50% (3). As a chronic ocular condition, myopia may act as a prolonged psychosocial stressor during its onset and development (4). Stressful events in early life can affect later responses to other stressors (5). It is related to unhealthy habits including reduced physical activity, increased television viewing, and increased smoking and alcohol consumption (6, 7). Prolonged stress affects homeostasis and increases the risk of developing mental illnesses (5, 8). The association between myopia and mental health has garnered considerable interest.

Myopic blurred vision can hinder children's academic life and daily activities. In a historical perspective, Seitler conceptualized myopia as a tense, unconscious defense mechanism that causes extraocular muscles to tighten, thereby leading to discontinuities in the process of separation-individuation (9). This disruption contributed to separation anxiety and a diminished ability to cope with the external world (9). The incidence of myopia is rising rapidly among younger children (10). Their physical and neurobehavior is undergoing a significant development (11). As visual strain, during the adaptation to correction, myopia inevitably causes abnormal psychological response (12). Myopia is incurable and requires long-term correction, with progression typically persisting throughout childhood and adolescence (13). Patients with chronic diseases receiving long-term treatment are prone to mental problems (14–16). Additionally, children face mental health factors such as sleep patterns, physical activity, and dietary habits, with the diagnosis of myopia further adding to psychological burdens (17, 18). We hypothesize that children diagnosed with myopia will exhibit significantly higher levels of emotional or behavioral difficulties compared to emmetropic peers.

Current studies on myopia and mental health rarely focused on children and psychological risk factors were not adequately isolated (19–22). Several studies found that myopic children exhibited poorer mental health, and faced challenges in learning when their myopia remained uncorrected (23–25). However, these studies primarily examined children in grades 3 and 4 in rural areas, neglecting urban populations and younger or older children, thereby limiting the generalizability of their findings. A similar study was conducted among middle school students in grades 7 and 9, but it relied on self-report rather than clinical examination to determine whether myopia or not, reducing the reliability of their findings (26). Li et al. (27) explored differences in anxiety and depression rates between myopic and emmetropic students, while they did not control for important psychological and environmental factors such as sociodemographic background and parental education. Overall, most existing studies are constrained by a narrow age range or insufficient control of psychological risk factors. There is an urgent need for more comprehensive studies, covering a wider age range and systematically considering potential confounders, to obtain stronger evidence on the association between childhood myopia and mental health.

Zibo, located in the central of Shandong Province, China. In 2022, the per capita gross domestic product (GDP) stood at 96,938 yuan, and the ratio of general practitioners to the permanent population was 3.23 per 10,000. This region's economic development is at the country's forefront, and its medical facilities are relatively sophisticated (28, 29). The prevalence of myopia among children and adolescents in Shandong was recorded at 58.66%, which is close to the national average prevalence rate (30). To better understand the association between childhood myopia and mental health, a cross-sectional study was embarked within the 9-year compulsory education system, providing insights that may be applicable to other regions with similarly high rates of myopia.

## 2 Methods

### 2.1 Study design and participants

This cross-sectional study was executed from September to October in 2023. One urban and one rural school were randomly selected via whole-cluster sampling from 14 schools at compulsory education system in Huantai County, Zibo City. The Wenjuanxing platform was used for data collection (https://www.wjx.cn/app/survey.aspx). Within the scope of the school's eye examinations, children were provided with specific questionnaire instructions, which they subsequently completed by scanning a QR code using parent's smartphone at post-school hours, under the supervision of their parent. The inclusion criteria were delineated as follows: (1) both the child and his/her parents agreed to participate in the eye examinations, (2) the child was not afflicted with any major physical or mental illness, and (3) both the child and his/her parents agreed to participate in the questionnaire survey. The exclusion criteria were delineated as follows: (1) children did not complete eye examinations including cycloplegic autorefraction, and the lack of eye examination data could affect the judgement of refractive status, (2) children were diagnosed with major illnesses, which could affect their mental health, (3) children with hyperopia, which might affect near visual acuity, and (4) children did not complete the questionnaire, or the questionnaire was abnormal (such as inconsistent and confusing answers).

Drawing from a national study, the likelihood of Chinese children and adolescents aged 6–16 years exhibiting behavioral and emotional problems was determined to be 17.6%, a two-sided, 0.05 significance level test with a 80% statistical power, leading to a calculated sample size of 496 (31).

**Figure 1:**
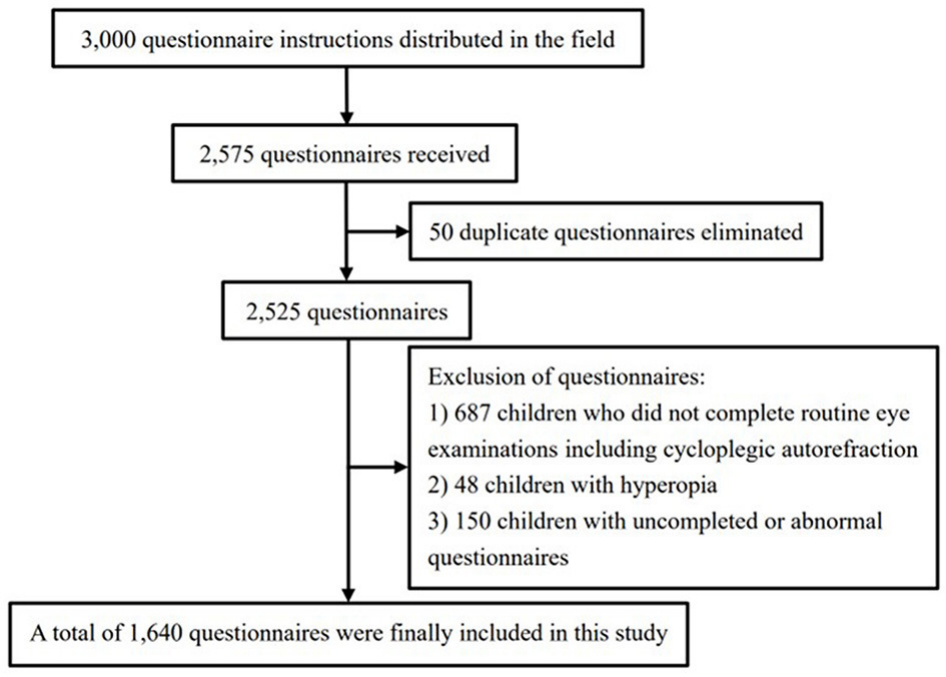
Flow diagram of questionnaire distribution and collation.

A total of 3,000 questionnaire instructions were distributed at school, and 2,575 questionnaires were received. Initially, it was observed that 50 children submitted the questionnaire on two occasions. Consequently, only the questionnaire with the superior level of integrity was retained. Secondly, the questionnaire data were merged with the eye examination data based on school, class, and name information. This process identified 687 children who had not undergone routine eye examinations, including cycloplegic autorefraction. These children were subsequently excluded from the study. Thirdly, based on the eye examination data, the myopic and emmetropic children were retained, while 48 children were excluded due to a diagnosis of hyperopia. Fourthly, 150 children were identified as uncompleted or abnormal questionnaires. This included 3 children with uncompleted questionnaire, 10 children with abnormal parental ages, 37 children with unusual sleeping time, 23 children with abnormal SDQ scale information, and 77 children were diagnosed with emmetropia but lying about declining visual acuity in the questionnaire. The aforementioned children were excluded from the study. Ultimately, the study included 1,640 questionnaires, which exceeded the initially calculated sample size (see Figure 1).

### 2.2 Eye examinations and visual acuity surveys

Initially, all children underwent a slit lamp examination and funduscopic examination, conducted by two seasoned ophthalmologists. Subsequently, a comprehensive series of visual acuity examinations were carried out by professional optometrists. These included naked-eye visual acuity tests at a standard testing distance of 4 m, based on the early treatment diabetic retinopathy study (ETDRS) Log MAR E vision chart (Precision Vision, Villa Park, Illinois, USA), non-contact tonometry (Topcon CT80; Topcon Corp., Tokyo, Japan), and both non-cycloplegic and cycloplegic autorefraction (Nidek ARK-1, CO., LTD, Japan). This study used 1% cyclopentolate hydrochloride Ophthalmic Solution (Alcon, Fort Worth, TX, USA) as the cycloplegic eye drops (32). The drops were dispensed at a regimen of 1 drop every 5 min, totaling 3 doses (32). Post-administration, children were directed to rest with their eyes closed for a duration of 30 min. Subsequently, observations were made regarding the pupillary light reflex and diameter. In instances where the pupillary reflex disappeared or the pupil diameter exceeded 6 mm, an autorefraction test was employed. If the pupil diameter measured less than 6 mm, the eye drops were reapplied. Following a 10-min interval, the autorefraction test was carried out.

The spherical equivalence (SE) is computed by summing the spherical refractive error and half of the cylindrical refractive error. As for refraction status, myopia is defined as SE post-cycloplegia ≤ -0.50 D, while emmetropia is defined as one in which children are not affected by loss of distance visual acuity (−0.50 D ≤ SE post-cycloplegia ≤ 2.00 D) (2). Additionally, children were asked about any alterations in their visual acuity over the past year via questionnaire, which required a simple “yes” or “no” response to a question, “Did you experience a decline in visual acuity last year?”

### 2.3 Assessment of mental health

The strengths and difficulties questionnaire (SDQ) was employed as the primary tool for evaluating children's mental health problems in this study (33). The SDQ is a universally recognized instrument for screening children about potential mental health problems (34–37). The Chinese self-report version of the SDQ, designed for children aged 4–17 years, boasts robust measurement properties, including a high degree of internal consistency (Cronbach's alpha coefficient = 0.81) and reliability (Pearson's correlation coefficient = 0.71) (38). The SDQ is partitioned into five subscales: emotional symptoms, conduct problems, hyperactivity/inattention, peer relationship problems, and prosocial behavior. It comprises 25 items, each scored on a three-point scale (0 = not true, 1 = somewhat true, 2 = completely true). All subscales, with the exception of prosocial behavior, can be aggregated to derive a SDQ total difficulties score that ranges from 0 to 40, where a higher score signifies poorer mental health. Goodman's criteria (http://www.sdqinfo.com) were utilized to categorize children's mental health status into normal (SDQ total difficulties score ≥0 and ≤ 15), borderline (SDQ total difficulties score ≥16 and ≤ 19), and abnormal (SDQ total difficulties score ≥20), employing 16 and 20 as the cut-off values. In this study, the Cronbach's alpha coefficients of the SDQ and its subscales are 0.687, 0.662, 0.249, 0.725, 0.231, and 0.747.

### 2.4 Covariates information

A range of covariates were selected for this study based on previous research, including covariates answered by children: gender (boy/girl), age (years), location of school (urban school/rural school), number of friends (<3/3–5/>5), school bullying (no/yes), academic requirements (low/high), academic performance (below average/average/above average), exercise time per week (<1 h/1–5 h/5–10 h/>10 h), and sleeping time per day (hours); covariates answered by parents: family type (with only one child/with more than one child), average age of parents (years), marital status of parents (normal/others), parental education level (primary or secondary school/high or vocational high school/junior college, university or above), and total income per year (Chinese Yuan) (<30,000/30,000–60,000/60,000–90,000/90,000–120,000/>120,000), and resident population (<4/4/>4) (18, 39–44).

### 2.5 Ethics approval and consent to participate

This study involving human participants followed the Declaration of Helsinki, and was reviewed and approved by the Ethics Committee of the Affiliated Eye Hospital of Shandong University of Traditional Chinese Medicine (HEC-KS-202305KY). Written informed consent to participate in this study was provided to the legal parents/next of kin of the participants.

### 2.6 Statistical analyses

Continuous variables were expressed as mean (standard deviation) and categorical variables were expressed as numbers (percentage). The Kruskal–Wallis test and Chi-Square test were applied to compare the differences between children with normal mental health status, borderline mental health status, and abnormal mental health status. Multiple linear regression analysis model for continuous outcome (SDQ total difficulties score) and logistic regression model for binary outcome (abnormal mental health problems and borderline mental health problems) were used to investigate the association between the characteristics of myopia and children's mental health. Firstly, the analysis began with the crude model. Then, a series of factors including gender, age, location of school, number of friends, school bullying, academic requirements, academic performance, exercise time per week, sleeping time per day, family type, average age of parents, marital status of parents, parental education level, total income per year, and resident population was adjusted.

The association between myopia and mental health was further investigated among children across various grades, and multiple linear regression analysis model was performed separately on four subgroups, each corresponding to different grades. First, the analysis began with the crude model. Subsequently, adjustments for the aforementioned covariates were adjusted.

In an effort to bolster the validity of the conclusions, the refractive status of children who had undergone routine eye examinations, but had not participated in cycloplegic autorefraction, was assessed using data from non-cycloplegic autorefraction, and then the statistical analyses was performed among all children with autorefraction. The outcomes of these analyses were shown in the Supplementary material.

All statistical analyses were executed using the SPSS software, version 27.0. The Akaike's information criterion (AIC) was employed to evaluate the goodness of fit of the model. A *P*-value of less than 0.05 for a two-sided test was deemed to denote statistical significance.

## 3 Results

### 3.1 Sample characteristics

The characteristics of the participants are presented in Table 1. Among all children, 836 (50.98%) children were boys, the mean (SD) age was 9.39 (2.22) years old, and age ranged from 6 to 16 years. 1,531 (93.35%) children exhibited a normal mental health status, 91 (5.55%) children exhibited a borderline mental health status, and 18 (1.10%) children exhibited an abnormal mental health status. The distribution of myopic children was 43.04% among those with normal mental health status, 51.65% among those with borderline mental health status, and 77.78% among those with abnormal mental health status. The prevalence of children who reported a decline in visual acuity last year was 27.89% among those with normal mental health status, 42.86% among those with borderline mental health status, and 44.44% among those with abnormal mental health status.

**Table 1:** Characteristics of participants in the study.

**Characteristics**	**Total (*N* = 1,640)**	**Children with normal mental health status (*N* = 1,531)**	**Children with borderline mental health status (*N* = 91)**	**Children with abnormal mental health status (*N* = 18)**	***P*-value^a^**
**Children's characteristics**					
**Gender**					
Boy	836 (50.98%)	767 (50.10%)	57 (62.64%)	12 (66.67%)	**0.027**
Girl	804 (49.02%)	764 (49.90%)	34 (37.36%)	6 (33.33%)	
**Age, years (sd)**	9.39 (2.22)	9.35 (2.19)	9.93 (2.54)	10.78 (2.37)	**0.003**
**Location of school**					
Urban school	834 (50.85%)	777 (50.75%)	46 (50.55%)	11 (61.11%)	0.681
Rural school	806 (49.15%)	754 (49.25%)	45 (49.45%)	7 (38.89%)	
**ETDRS (sd)**	75.23 (10.60)	75.35 (10.51)	74.31 (11.10)	69.72 (14.12)	0.148
**Refraction status**					
Emmetropia	920 (56.10%)	872 (56.96%)	44 (48.35%)	4 (22.22%)	**0.004**
Myopia	720 (43.90%)	659 (43.04%)	47 (51.65%)	14 (77.78%)	
**Visual acuity decline last year** ^b^					
No	1,166 (71.10%)	1,104 (72.11%)	52 (57.14%)	10 (55.56%)	**0.003**
Yes	474 (28.90%)	427 (27.89%)	39 (42.86%)	8 (44.44%)	
**Number of friends**					
< 3	116 (7.07%)	107 (6.99%)	8 (8.79%)	1 (5.56%)	0.803
3–5	686 (41.83%)	636 (41.54%)	41 (45.05%)	9 (50.00%)	
>5	838 (51.10%)	788 (51.47%)	42 (46.15%)	8 (44.44%)	
**School bullying**					
No	1,615 (98.48%)	1,515 (98.95%)	83 (91.21%)	17 (94.44%)	**< 0.001**
Yes	25 (1.52%)	16 (1.05%)	8 (8.79%)	1 (5.56%)	
**Academic requirements**					
Low	680 (41.46%)	629 (41.08%)	45 (49.45%)	6 (33.33%)	0.226
High	960 (58.54%)	902 (58.92%)	46 (50.55%)	12 (66.67%)	
**Academic performance**					
Below average	480 (29.27%)	416 (27.17%)	51 (56.04%)	13 (72.22%)	**< 0.001**
Average	771 (47.01%)	739 (48.27%)	30 (32.97%)	2 (11.11%)	
Above average	389 (23.72%)	376 (24.56%)	10 (10.99%)	3 (16.67%)	
**Exercise time per week**					
< 1 h	336 (20.49%)	301 (19.66%)	28 (30.77%)	7 (38.89%)	**0.025**
1–5 h	756 (46.10%)	705 (46.05%)	44 (48.35%)	7 (38.89%)	
5–10 h	352 (21.46%)	336 (21.95%)	14 (15.38%)	2 (11.11%)	
>10 h	196 (11.95%)	189 (12.34%)	5 (5.49%)	2 (11.11%)	
**Sleeping time per day, hours (sd)**	9.11 (1.09)	9.14 (1.06)	8.65 (1.39)	8.39 (1.61)	**< 0.001**
**Family's characteristics**					
**Family type**					
With only one child	255 (15.55%)	231 (15.09%)	23 (25.27%)	1 (5.56%)	**0.017**
With more than one child	1,385 (84.45%)	1,300 (84.91%)	68 (74.73%)	17 (94.44%)	
**Average age of parents, year (sd)**	38.35 (5.04)	38.39 (5.04)	37.67 (5.07)	39.17 (4.98)	0.403
**Marital status of parents**					
Normal	1,534 (93.54%)	1,439 (93.99%)	80 (87.91%)	15 (83.33%)	**0.015**
Others	106 (6.46%)	92 (6.01%)	11 (12.09%)	3 (16.67%)	
**Parental education level**					
Primary or secondary school	572 (34.88%)	526 (34.36%)	40 (43.96%)	6 (33.33%)	0.468
High or vocational high school	630 (38.41%)	592 (38.67%)	31 (34.07%)	7 (38.89%)	
Junior college/university or above	438 (26.71%)	413 (26.98%)	20 (21.98%)	5 (27.78%)	
**Total income per year, Chinese Yuan**					
< 30000	246 (15.00%)	221 (14.44%)	23 (25.27%)	2 (11.11%)	0.104
30000–60000	474 (28.90%)	444 (29.00%)	22 (24.18%)	8 (44.44%)	
60000–90000	343 (20.91%)	319 (20.84%)	21 (23.08%)	3 (16.67%)	
90000–120000	305 (18.60%)	290 (18.94%)	14 (15.38%)	1 (5.56%)	
>120000	272 (16.59%)	257 (16.79%)	11 (12.09%)	4 (22.22%)	
**Resident population**					
< 4	302 (18.41%)	273 (17.83%)	26 (28.57%)	3 (16.67%)	0.144
4	754 (45.98%)	711 (46.44%)	35 (38.46%)	8 (44.44%)	
>4	584 (35.61%)	547 (35.73%)	30 (32.97%)	7 (38.89%)	

^**a**^Continues variables were compared by Kruskal–Wallis test; categorical variables were compared by Chi-Square test.

^**b**^Adoption by self-reporting.

The bold values represent the results that are statistically significant.

Comparison between groups showed that, children exhibiting psychological abnormalities were more likely to be boy, be older, be myopic, report a decline in visual acuity last year, suffer from school bullying, be worse in academic performance, have less exercise time, sleep less, have a family type with more than one child, and have parents with an abnormal marital status. However, no significant differences were observed among the three subgroups (normal, borderline, and abnormal) with respect to other characteristics such as location of school, ETDRS score, number of friends, academic requirements, average age of parents, parental education level, total income per year, and resident population.

In terms of refractive status, 56.10% of children were emmetropic, whereas 43.90% of children were myopic. The mean ETDRS score (SD) was 75.23 ± 10.60. Regarding the decline in visual acuity last year, 71.10% of children reported no decline, while 28.90% reported experiencing a decline in visual acuity.

### 3.2 Visual acuity decline associated with poorer mental health conditions

In the multiple linear regression analysis, after accounting for covariates (see Table 2), this study found that children who reported a decline in visual acuity last year had a significantly higher SDQ total difficulties score (β = 0.564, 95% CI: 0.064, 1.064; *P* = 0.027) and poorer mental health conditions compared to their counterparts who did not report such a decline.

**Table 2:** Association between characteristics of myopia and SDQ total difficulties score of children.

**Characteristics of myopia**	**Crude model**		**Adjusted model** ^**a**^	
	β **(95%CI)**	***P*** **-value**	β **(95%CI)**	***P*** **-value**
**Refraction status**				
Emmetropia	ref	ref	ref	ref
Myopia	0.326 (−0.123, 0.774)	0.154	0.148 (−0.330, 0.626)	0.544
**ETDRS**	−0.008 (−0.029, 0.013)	0.453	−0.004 (−0.024, 0.015)	0.660
**Visual acuity decline last year** ^b^				
No	ref	ref	ref	ref
Yes	**0.740 (0.250, 1.230)**	**0.003**	**0.564 (0.064, 1.064)**	**0.027**

^**a**^Aadjusting for gender, age, location of school, number of friends, school bullying, academic requirements, academic performance, exercise time per week, sleeping time per day, family type, average age of parents, marital status of parents, parental education level, total income per year, resident population.

^**b**^Adoption by self-reporting.

The bold values represent the results that are statistically significant.

### 3.3 Myopia and visual acuity decline associated with increased mental health risks

In the binary logistic regression analysis, after accounting for covariates (see Table 3), this study found that myopic children exhibited a significantly higher risk of abnormal mental health problems compared to emmetropic children (OR = 3.601, 95% CI: 1.070, 12.456; *P* = 0.039). As the number of correctly identified visual markers on the ETDRS visual acuity scale increased, there was a corresponding decrease in the risk of abnormal mental health problems among children (OR = 0.964, 95% CI: 0.933, 0.997; *P* = 0.033). Furthermore, children who reported a decline in visual acuity last year were found to be at a higher risk of borderline mental health problems compared to those who did not report such a decline (OR = 1.863, 95% CI: 1.135, 3.057; *P* = 0.014).

**Table 3:** Association between characteristics of myopia and mental health problems of children.

**Characteristics of myopia**	**Abnormal mental health problems**				**Borderline mental health problems**			
	**Crude model**		**Adjusted model** ^a^		**Crude model**		**Adjusted model** ^a^	
	**OR (95% CI)**	***P*** **-value**	**OR (95% CI)**	***P*** **-value**	**OR (95% CI)**	***P*** **-value**	**OR (95% CI)**	***P*** **-value**
**Refraction status**								
Emmetropia	Ref	Ref	Ref	Ref	Ref	Ref	Ref	Ref
Myopia	**4.631 (1.517, 14.135)**	**0.007**	**3.601 (1.070, 12.456)**	**0.039**	1.413 (0.926, 2.159)	0.109	1.243 (0.759, 2.035)	0.388
**ETDRS**	**0.964 (0.933, 0.996)**	**0.026**	**0.964 (0.933, 0.997)**	**0.033**	0.991 (0.973, 1.010)	0.359	0.992 (0.972, 1.013)	0.449
**Visual acuity decline last year** ^b^								
No	Ref	Ref	Ref	Ref	Ref	Ref	Ref	Ref
Yes	2.068 (0.811, 5.276)	0.128	1.430 (0.500, 4.088)	0.504	**1.939 (1.261, 2.981)**	**0.003**	**1.863 (1.135, 3.057)**	**0.014**

^**a**^Adjusting for gender, age, location of school, number of friends, school bullying, academic requirements, academic performance, exercise time per week, sleeping time per day, family type, average age of parents, marital status of parents, parental education level, total income per year, resident population.

^**b**^Adoption by self-reporting.

The bold values represent the results that are statistically significant.

### 3.4 Myopia and visual acuity decline linked to poorer mental health in grades 3-4

In the multiple linear regression analyses of subgroups, after accounting for covariates (see Table 4), this study found that myopic children in the grades 3-4 subgroup (9–10 years old) had a significantly higher SDQ total difficulties score (β = 1.065, 95% CI: 0.200, 1.929; *P* = 0.016) and poorer mental health compared to their emmetropic counterparts. Additionally, children who reported a decline in visual acuity last year had a higher SDQ total difficulties score (β = 0.981, 95% CI: 0.011, 1.951; *P* = 0.048) and poorer mental health compared to those who did not report such a decline.

**Table 4:** Association between characteristics of myopia and SDQ total difficulties score of children in different subgroups.

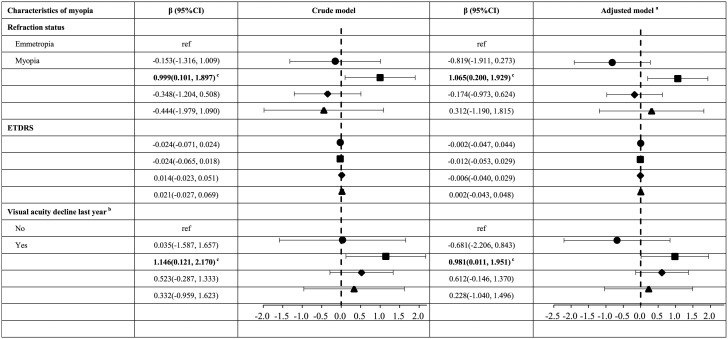

^**a**^Adjusting for gender, age, location of school, number of friends, school bullying, academic requirements, academic performance, exercise time per week, sleeping time per day, family type, average age of parents, marital status of parents, parental education level, total income per year, resident population.

^**b**^Adoption by self-reporting.

^**c**^*P* < 0.05.

• Subgroup 1: grade1-2, ■ subgroup 2: grade3-4, ♦ subgroup 3: grade5-6, ▴ subgroup 4: grade7-8.

The bold values represent the results that are statistically significant.

## 4 Discussion

Primary and secondary school represent stages that are particularly sensitive to the onset and development of myopia, with the prevalence of this condition escalating in a stepwise fashion across grades. The emergence of myopia and alterations in visual acuity constitute stressful events that students are required to manage. Therefore, understanding this association would be advantageous for protection of children's mental health. This study explored the association between myopia and mental health, employing the strengths and difficulties questionnaire (SDQ) as a tool. Additional subgroup analyses was conducted to explore the differences among children across various grades. A negative association between myopia and mental health was found among primary and secondary school students, particularly pronounced in grades 3-4.

### 4.1 Myopia as a risk factor for childhood mental health

This study identified myopia as a risk factor for mental health problems in children. Children who reported a decline in visual acuity last year exhibited higher SDQ scores and poorer mental health. This observation aligns with a previous study by Yi et al. (24) where they found a correlation between a line decline in visual acuity and a 0.25 standard deviation increase in the standardized mental health test (MHT) score. A study based on the National Health and Nutrition Examination Survey (NHANES) found a significant association between self-reported visual function loss and depression (45). No significant result was found in refractive status and ETDRS. This could potentially be attributed to the ages, grades, and durations of myopia among children in this study. Huang et al. (26) reported that the adverse effects of myopia were more pronounced in older students, who were dealing with more severe myopia and depression. The average age in borderline mental health status group was 9.93 years old, while in the abnormal mental health status group, it was 10.78 years. This suggested that a higher proportion of students in the borderline mental health status group is in lower grades, and a greater number of students in the abnormal mental health status group is in higher grades. The results of the binary logistic regression analyses verified the phenomenon that no significant results were found in the multiple linear regression analyses regarding refractive status and ETDRS. Myopic children were 3.601 times more likely to exhibit abnormal mental health problems compared to emmetropic children. It was less likely to exhibit abnormal mental health problems with an increase in the number of correctly identified visual markers on the ETDRS chart. Zhang et al. (21) and Li et al. (27) found a significant association between myopia and mental health problems, specifically anxiety, among Chinese high school and university freshmen, respectively. Ding et al. (46) found that among primary school students, compared with children with normal vision, myopic children had worse mental health overall. No significant association for refractive status and ETDRS was found while comparing normal and borderline mental health groups. In this study, myopic children with borderline mental health conditions were younger who tended to have less severe myopia (13). According to Yi et al. (24), standardized MHT scores increased progressively as vision impairment worsened: by 0.08 standard deviations for mild poor vision, 0.16 for moderate, and 0.32 for severe, compared to students with normal vision. This result suggested that for children with mild myopia, differences in mental health outcomes are not as pronounced. Children who reported a decline in visual acuity last year were found to be at a higher risk of borderline mental health problems compared to those who did not report such a decline. The overall myopia rate among children increased rapidly in the early stage (6–10 years old), and the grow of the prevalence rate slowed down with age (3). For lower grades children, it's a new experience transiting from a non-myopic to a myopic state. It will result in borderline mental health problems. Both refractive status and ETDRS serve as indicators of the degree of visual acuity, and the decline in visual acuity last year reported by children reflects changes in their visual acuity. Thus, this study identified significant results in both refractive status and ETDRS in the normal vs. abnormal group, and in visual acuity decline in the normal vs. borderline group. What's more, survey data obtained from non-cycloplegic autorefraction eye examinations of children was integrated into the original framework. The outcomes were in line with these findings based on children who underwent cycloplegic autorefraction (see Supplementary material).

### 4.2 Age-specific vulnerability: myopia and mental health in grades 3-4

Myopic children, especially those in grades 3-4, exhibited poorer mental health. These children, typically aged 8–10, are particularly vulnerable to myopia due to the rapid growth of the eye axis during this developmental stage (47). The increased time spent on close-up studying, combined with reduced outdoor activity, accelerates the onset and progression of myopia (48). Myopia can be a stressful event for them, especially when it disrupts daily activities and cognitive functions (49). This phenomenon is even more pronounced due to the fact that younger children begin to establish their identity and social status (50, 51). In subgroup analyses based on grade, a significant association between myopia and visual acuity decline and poorer mental health was found in the grades 3-4 subgroup. Similarly, Yi et al. (24) found a significant positive association between poor vision and deteriorating mental health in grades 4 and 5. Given the vulnerability of this age group, it is essential to raise awareness about myopia early on, ensuring children do not panic at the onset of vision problems. Regular eye examinations and timely corrective measures are crucial for reducing the disruptions of daily activities and promoting adaptability during key developmental stages, supporting both their visual and mental health.

### 4.3 Implications

This study supports that we should not only focus on myopia prevention and control, but also pay attention to their mental health problems. Given the link between myopia and mental health, screening and intervention for mental health problems in children is critical. In addition to vision correction, a comprehensive approach such as encouraging outdoor activities, limiting near work time, and developing emotional resilience through counseling and peer support can reduce the psychological burden associated with myopia (52). The whole society should be called for paying attention to the mental health of children with myopia, so that they can grow up healthily.

### 4.4 Limitations

There were several limitations in this study. First, the scope of this study, confined to one urban and one rural 9-year school in a single region, limited the generalizability of these findings to other regions of China. Future research should contemplate conducting studies across multiple regions. Second, given that the questionnaire were completed by children under parental supervision, there may be reporting bias, and using both child- and parent-reported versions of the SDQ in future research is recommended. Third, all the children in grade 9 who participated in the routine eye examinations did not undergo cycloplegic autorefraction, primarily due to the pressures associated with further education. In light of this, additional analyses was conducted based on the results derived from non-cycloplegic autorefraction. Final, the cross-sectional nature of this study precluded us from establishing a causal relationship between myopia and mental health.

## 5 Conclusions

In summary, this study found that myopic children were more prone to exhibit mental health problems compared to emmetropic children, and children with superior visual acuity generally exhibited better mental health, particularly in grades 3-4. These findings highlight the importance of implementing proactive measures for myopia prevention and control to safeguard the visual health of children. Concurrently, the screening and intervention of mental health issues among myopic children constitute a critical task that warrants attention. This study provides contemporary epidemiological evidence on the association between childhood myopia and mental health, offering valuable insights for regions with a high prevalence of myopia. Future research aimed at exploring the impact of myopia on mental health will undoubtedly garner significant interest.

## Data Availability

The raw data supporting the conclusions of this article will be made available by the authors, without undue reservation.
