# Supplementary material for: The association between myopia and mental health among Chinese children in primary and secondary school: a cross-sectional study
Source: Front Public Health. 2025 Jun 3;13:1598790. doi: 10.3389/fpubh.2025.1598790 (PMC12170659; doi:10.3389/fpubh.2025.1598790)
Supplement: Supplementary file 1 [file Table_1.docx]

***Supplementary Material***

1. **Supplementary Figures and Tables**
   1. **Supplementary Figure**

3,000 questionnaire instructions distributed in the field

2,575 questionnaires received

50 duplicate questionnaires eliminated

2,525 questionnaires

Exclusion of questionnaires:

1) 148 children who did not complete routine eye examinations

2) 51 children with hyperopia

3) 178 children with unfilled or abnormal questionnaires

A total of 2,148 questionnaires were finally included in this study

Figure S1 Flow diagram of questionnaire distribution and collation

- 1. **Supplementary Tables**

**Table S1 Characteristics of all children with routine eye examinations in the study**

|  | **Total (N=2,148)** | **Children with normal mental health status (N=1,998)** | **Children with borderline mental health status (N=121)** | **Children with abnormal mental health status (N=29)** | ***P*-value ^a^** |
| --- | --- | --- | --- | --- | --- |
| **Children’s characteristics** |  |  |  |  |  |
| **Gender** |  |  |  |  | 0.317 |
| Boy | 1,075(50.05%) | 991(49.60%) | 68(56.20%) | 16(55.17%) |  |
| Girl | 1,073(49.95%) | 1,007(50.40%) | 53(43.80%) | 13(44.83%) |  |
| **Age, years (sd)** | 9.93(2.53) | 9.87(2.50) | 10.52(2.76) | 11.90(2.41) | **<0.001** |
| **Location of school** |  |  |  |  | 0.932 |
| Urban school | 1,028(47.86%) | 958(47.95%) | 57(47.11%) | 13(44.83%) |  |
| Rural school | 1,120(52.14%) | 1,040(52.05%) | 64(52.89%) | 16(55.17%) |  |
| **ETDRS (sd)** | 75.08(10.99) | 75.25(10.77) | 73.83(12.59) | 69.00(16.10) | 0.092 |
| **Refraction status** |  |  |  |  | **0.002** |
| Emmetropia | 1,025(47.72%) | 969(48.50%) | 51(42.15%) | 5(17.24%) |  |
| Myopia | 1,123(52.28%) | 1,029(51.50%) | 70(57.85%) | 24(82.76%) |  |
| **Visual acuity decline last year ^b^** |  |  |  |  | **<0.001** |
| No | 1,407(65.50%) | 1,328(66.47%) | 68(56.20%) | 11(37.93%) |  |
| Yes | 741(34.50%) | 670(33.53%) | 53(43.80%) | 18(62.07%) |  |
| **Number of friends** |  |  |  |  | 0.196 |
| <3 | 149(6.94%) | 136(6.81%) | 12(9.92%) | 1(3.45%) |  |
| 3-5 | 864(40.22%) | 794(39.74%) | 55(45.45%) | 15(51.72%) |  |
| >5 | 1,135(52.84%) | 1,068(53.45%) | 54(44.63%) | 13(44.83%) |  |
| **School bullying** |  |  |  |  | **<0.001** |
| No | 2,115(98.46%) | 1,976(98.90%) | 112(92.56%) | 27(93.10%) |  |
| Yes | 33(1.54%) | 22(1.10%) | 9(7.44%) | 2(6.90%) |  |
| **Academic requirements** |  |  |  |  | 0.146 |
| Low | 973(45.30%) | 908(45.45%) | 57(47.11%) | 8(27.59%) |  |
| High | 1,175(54.70%) | 1,090(54.55%) | 64(52.89%) | 21(72.41%) |  |
| **Academic performance** |  |  |  |  | **<0.001** |
| Below average | 510(23.74%) | 432(21.62%) | 62(51.24%) | 16(55.17%) |  |
| Average | 901(41.95%) | 856(42.84%) | 40(33.06%) | 5(17.24%) |  |
| Above average | 737(34.31%) | 710(35.54%) | 19(15.70%) | 8(27.59%) |  |
| **Exercise time per week** |  |  |  |  | **0.003** |
| <1 hour | 424(19.74%) | 378(18.92%) | 34(28.10%) | 12(41.38%) |  |
| 1-5 hours | 987(45.95%) | 918(45.95%) | 57(47.11%) | 12(41.38%) |  |
| 5-10 hours | 483(22.49%) | 456(22.82%) | 24(19.83%) | 3(10.34%) |  |
| >10 hours | 254(11.82%) | 246(12.31%) | 6(4.96%) | 2(6.90%) |  |
| **Sleeping time per day, hours (sd)** | 8.98(1.11) | 9.03(1.07) | 8.49(1.36) | 8.17(1.39) | **<0.001** |
| **Family’s characteristics** |  |  |  |  |  |
| **Family type** |  |  |  |  | **0.005** |
| With only one child | 343(15.97%) | 310(15.52%) | 31(25.62%) | 2(6.90%) |  |
| With more than one child | 1,805(84.03%) | 1,688(84.48%) | 90(74.38%) | 27(93.10%) |  |
| **Average age of parents, year (sd)** | 38.88(5.25) | 38.87(5.24) | 38.54(5.15) | 40.79 (6.16) | 0.180 |
| **Marital status of parents** |  |  |  |  | **0.035** |
| Normal | 2,042(95.07%) | 1,906(95.40%) | 110(90.91%) | 26(89.66%) |  |
| Others | 106(4.93%) | 92(4.60%) | 11(9.09%) | 3(10.34%) |  |
| **Parental education level** |  |  |  |  | 0.120 |
| Primary or secondary school | 796(37.06%) | 726(36.34%) | 58(47.93%) | 12(41.38%) |  |
| High or vocational high school | 808(37.62%) | 757(37.89%) | 40(33.06%) | 11(37.93%) |  |
| Junior college/university or above | 544(25.33%) | 515(25.78%) | 23(19.01%) | 6(20.69%) |  |
| **Total income per year, Chinese Yuan** |  |  |  |  | **0.013** |
| <30000 | 333(15.50%) | 296(14.81%) | 29(23.97%) | 8(27.59%) |  |
| 30000-60000 | 605(28.17%) | 560(28.03%) | 33(27.27%) | 12(41.38%) |  |
| 60000-90000 | 465(21.65%) | 432(21.62%) | 29(23.97%) | 4(13.79%) |  |
| 90000-120000 | 393(18.30%) | 376(18.82%) | 16(13.22%) | 1(3.45%) |  |
| >120000 | 352(16.39%) | 334(16.72%) | 14(11.57%) | 4(13.79%) |  |
| **Resident population** |  |  |  |  | 0.148 |
| <4 | 416(19.37%) | 378(18.92%) | 34(28.10%) | 4(13.79%) |  |
| 4 | 975(45.39%) | 913(45.70%) | 48(39.67%) | 14(48.28%) |  |
| >4 | 757(35.24%) | 707(35.39%) | 39(32.23%) | 11(37.93%) |  |

**^a^** continues variables were compared by Kruskal–Wallis test; categorical variables were compared by Chi-Square test

**^b^** adoption by self-reporting

**Table S2 Association between characteristics of myopia and SDQ total difficulties score of children**

| **Characteristics of myopia** | **Crude model** | |  | **Adjusted model ^a^** | |
| --- | --- | --- | --- | --- | --- |
|  | **β (95%CI)** | ***P*-value** |  | **β (95%CI)** | ***P*-value** |
| **Refraction status** |  |  |  |  |  |
| Emmetropia | ref | ref |  | ref | ref |
| Myopia | 0.227(-0.167, 0.620) | 0.259 |  | 0.060(-0.372, 0.491) | 0.786 |
| **ETDRS** | -0.012(-0.030, 0.005) | 0.173 |  | -0.002(-0.019, 0.015) | 0.848 |
| **Visual acuity decline last year ^b^** |  |  |  |  |  |
| No | ref | ref |  | ref | ref |
| Yes | **0.718(0.305, 1.130)** | **0.001** |  | **0.613(0.191, 1.036)** | **0.004** |

**^a^** adjusting for gender, age, location of school, number of friends, school bullying, academic requirements, academic performance, exercise time per week, sleeping time per day, family type, average age of parents, marital status of parents, parental education level, total income per year, resident population

**^b^** adoption by self-reporting

**Table S3 Association between characteristics of myopia and mental health problems of children**

| **Characteristics of myopia** | **Abnormal mental health problems** | | | | |  | **Borderline mental health problems** | | | | |
| --- | --- | --- | --- | --- | --- | --- | --- | --- | --- | --- | --- |
|  | **Crude model** | |  | **Adjusted model ^a^** | |  | **Crude model** | |  | **Adjusted model ^a^** | |
|  | **OR（95%CI）** | ***P*-value** |  | **OR（95%CI）** | ***P*-value** |  | **OR（95%CI）** | ***P*-value** |  | **OR（95%CI）** | ***P*-value** |
| **Refraction status** |  |  |  |  |  |  |  |  |  |  |  |
| Emmetropia | Ref | Ref |  | Ref | Ref |  | Ref | Ref |  | Ref | Ref |
| Myopia | **4.520(1.718, 11.894)** | **0.002** |  | **3.001(1.014, 8.878)** | **0.047** |  | 1.293(0.892, 1.874) | 0.176 |  | 1.153(0.740, 1.797) | 0.529 |
| **ETDRS** | **0.963(0.940, 0.987)** | **0.002** |  | **0.967(0.941, 0.993)** | **0.014** |  | 0.989(0.974, 1.005) | 0.166 |  | 0.994(0.977, 1.010) | 0.451 |
| **Visual acuity decline last year ^b^** |  |  |  |  |  |  |  |  |  |  |  |
| No | Ref | Ref |  | Ref | Ref |  | Ref | Ref |  | Ref | Ref |
| Yes | **3.243(1.523, 6.906)** | **0.002** |  | **2.385(1.007, 5.650)** | **0.048** |  | **1.545(1.066, 2.239)** | **0.022** |  | 1.449(0.948, 2.215) | 0.087 |

**^a^** adjusting for gender, age, location of school, number of friends, school bullying, academic requirements, academic performance, exercise time per week, sleeping time per day, family type, average age of parents, marital status of parents, parental education level, total income per year, resident population

**^b^** adoption by self-reporting

**Table S4 Association between characteristics of myopia and SDQ total difficulties score of children in different subgroups**

| **Characteristics of myopia** | **β (95%CI)** | **Crude model** | **β (95%CI)** | **Adjusted model ^a^** |
| --- | --- | --- | --- | --- |
| **Refraction status** |  |  |  |  |
| Emmetropia | ref |  | ref |  |
| Myopia | -0.227(-1.224, 0.769) |  | -0.386(-1.319, 0.548) |  |
|  | 0.422(-0.395, 1.239) |  | 0.732(-0.041, 1.504) |  |
|  | -0.472(-1.263, 0.318) |  | -0.264(-1.000, 0.473) |  |
|  | -0.482(-1.718, 0.755) |  | 0.514(-0.680, 1.708) |  |
| **ETDRS** |  |  |  |  |
|  | -0.012(-0.057, 0.033) |  | 0.008(-0.036, 0.052) |  |
|  | -0.001(-0.038, 0.037) |  | -0.006(-0.031, 0.042) |  |
|  | 0.010(-0.024, 0.043) |  | 0.001(-0.030, 0.032) |  |
|  | 0.025(-0.058, 0.008) |  | -0.022(-0.053, 0.009) |  |
| **Visual acuity decline last year ^b^** |  |  |  |  |
| No | ref |  | ref |  |
| Yes | 0405(-1.005, 1.814) |  | 0.166(-1.167, 1.498) |  |
|  | 0.557(-0.350, 1.464) |  | 0.619(-0.227, 1.465) |  |
|  | 0.334(-0.403, 1.070) |  | 0.523(-0.168, 1.215) |  |
|  | 0.820(-0.058, 1.699) |  | **0.901(0.060, 1.742) ^c^** |  |
|  |  |  |  |  |
|  |  |  |  |  |

**^a^** adjusting for gender, age, location of school, number of friends, school bullying, academic requirements, academic performance, exercise time per week, sleeping time per day, family type, average age of parents, marital status of parents, parental education level, total income per year, resident population

**^b^** adoption by self-reporting

**^c^** *P*<0.05

●subgroup 1: grade1-2 ■subgroup 2: grade3-4 ◆subgroup 3: grade5-6 ▲subgroup 4: grade7-9
